# Impact of Substrate and Bright Resonances on Group Velocity in Metamaterial without Dark Resonator

**DOI:** 10.1038/srep14373

**Published:** 2015-09-23

**Authors:** Mohammad Parvinnezhad Hokmabadi, Ju-Hyung Kim, Elmer Rivera, Patrick Kung, Seongsin M. Kim

**Affiliations:** 1 Department of Electrical and Computer Engineering, The University of Alabama, Tuscaloosa, Alabama 35487, USA

## Abstract

Manipulating the speed of light has never been more exciting since electromagnetic induced transparency and its classical analogs led to slow light. Here, we report the manipulation of light group velocity in a terahertz metamaterial without needing a dark resonator, but utilizing instead two concentric split-ring bright resonators (meta-atoms) exhibiting a bright Fano resonance in close vicinity of a bright Lorentzian resonance to create a narrowband transmittance. Unlike earlier reports, the bright Fano resonance does not stem from an asymmetry of meta-atoms or an interaction between them. Additionally, we develop a method to determine the metamaterial “effective thickness”, which quantifies the influence of the substrate on the metamaterial response and has remained challenging to estimate so far. By doing so, very good agreement between simulated and measured group delays and velocities is accomplished. The proposed structure and method will be useful in designing optical buffers, delay lines, and ultra-sensitive sensors.

Manipulating the speed of light has been the focus of growing fundamental scientific interest, along with a high potential technological impact. Scientifically, braking photons and breaking the speed of light might literally bring about heavy photons^1^,^2^ and crucial challenges regarding causality^3^,^4^. In terms of technology, being able to stop light will lead to the development of optical buffers and delay lines as essential elements of future ultrafast all optical communication networks that could meet the ever-increasing demands for long-distance communications^5^,^6^. In addition, enhanced interaction of photons with matter by lowering the speed of light gives rise to reduced power consumption in nonlinear optical switching devices and ultra-accurate sensing performance of optical sensors^7^,^8^,^9^,^10^,^11^.

Most successful approaches to slow light rely on electromagnetic induced transparency (EIT), which is a quantum destructive interference phenomenon where a narrowband “dark” state opens up a transparency window inside the broader absorption band of a “bright” state^12^,^13^. EIT has been observed in various media including cold atoms, warm atoms, and plasmas, but cumbersome experimental conditions have often hampered its practical implementation^14^,^15^,^16^,^17^,^18^,^19^,^20^. Alternative approaches using classical analogues such as coupled resonators, photonic crystals and plasmonic structures are promising to bring EIT and slow light into real practical applications by relaxing experimental constraints^2^,^21^,^22^,^23^,^24^,^25^,^26^,^27^. Among them, plasmonic structures based on metamaterials have recently attracted great attention since metamaterials can be designed to mimic various quantum phenomena^28^,^29^,^30^,^31^. Several metamaterial structures have been developed to achieve broadband, multiband, actively controllable, and polarization insensitive EIT in order to slow down light in various spectra or even to obtain negative group velocities^32^,^33^,^34^,^35^,^36^,^37^,^38^,^39^,^40^,^41^,^42^,^43^. The vast majority of EIT metamaterials to date most closely mimic quantum destructive interference. In this type, dark meta-atoms are used to create a narrowband dark state that opens a transparency window within a broadband bright state arising from bright meta-atoms coupling directly to the electric field of an incident electromagnetic wave^32^,^33^,^34^,^35^,^36^,^37^,^38^,^39^,^44^,^45^,^46^. By contrast, there have been few reports of achieving EIT-like characteristics without using dark meta-atoms^40^,^41^. In this case, two bright meta-atoms directly couple to the incident electric field, leading to two resonances, (one of which being a Fano resonance) which are spectrally close to each other and where a narrowband transmission window emerges in between. In the less-than-a-handful of examples available to date, asymmetric SRRs or interaction between symmetric dipole rings have been reportedly thought to be necessary to achieve the Fano resonances in this type of metamaterials that give rise to EIT phenomenon^27^,^28^,^29^,^30^,^47^,^48^,^49^,^50^.

In this article, we report the observation of slow light phenomena in a terahertz metamaterial structure consisting of two concentric SRRs as bright resonators but without the need for a dark resonator. A sharp Fano resonance associated with the small SRR resides in spectral proximity of a Lorentzian resonance originating from the large SRR, which essentially results in a transparency window between the two resonances where the velocity of light is diminished. This new metamaterial structure demonstrates fast and negative light velocity at the resonance frequencies in addition to slow light in between. However, experimental determination of the group velocity of light in EIT-like metamaterials has remained challenging, by contrast to group delay, owing to the uncertainty in a fundamental parameter –the effective thickness of the metamaterial (t_m_)– needed in order to be able to match simulation results with experiments and which characterizes the influence of substrate on the response of the metamaterial^37^. Here, we report our novel approach to determine this parameter and obtain a fairly perfect agreement between simulations and experimental measurements for both group delays and group velocities. We then further study the effect of spectral separation between the Fano and Lorentzian resonances on slow light. Finally, we demonstrate that the sharp Fano resonance observed in our structure arises from excitation of the second order mode in a symmetric SRR without interaction with adjacent SRRs, unlike previously reported Fano metamaterials where breaking the symmetry of meta-atoms or interaction between adjacent symmetric meta-atoms were reportedly essential conditions to achieve Fano resonance.

## Results

### Design, Fabrication, and Characterization

Six metamaterial arrays (named R4C1, R4C3, R1C4, R1C2, R2C1, and R2C4) were designed and studied in this paper. The first three (R4C1, R4C3, and R1C4) are utilized to characterize group velocities and group delays, and to estimate the metamaterial effective thickness. The other three designs (R1C2, R2C1, and R2C4) are used to further analyze the effect of separation between bright resonances on slow light and to study the possible mechanisms behind our observations.

### Effective thickness of metamaterial and the impact of substrate on group velocity

**Figure 1:**
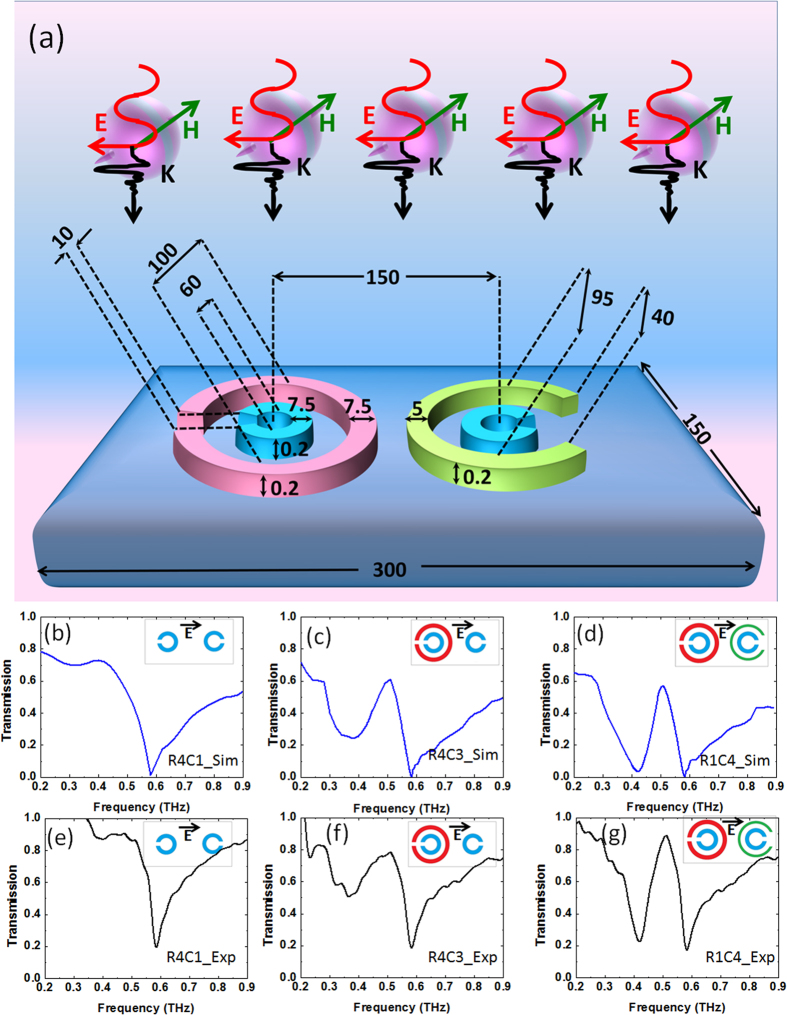
Schematic of Structure and absorption spectra. (**a**) A schematic illustration of R1C4 with dimensions all in micrometers, (**b–d**) are simulated transmission spectra and (**e–g**) are measured transmission spectra for samples R4C1, R4C3, and R1C4, respectively. Insets show front views of the corresponding structures.

A schematic representation of the unit cell of R1C4 is illustrated in Fig. 1a, and the front projection views of all three are displayed in the insets of Fig 1b–d. It is worthy to mention that all designs under study are essentially constructed from three different individual SRRs depicted using pink, blue, and green colors in the figures, such that any structure includes a combination of a few of these SRRs with either a 0° or 90° rotation. The dimensions in Fig. 1a are all in micrometers and will be kept the same in this entire study for all designs.

Comsol Multiphysics was utilized to simulate the response of these structures under an incident electromagnetic wave by using finite element method (FEM) (see Methods). We used periodic boundary conditions (PBC) for all side boundaries perpendicular to the plane of the SRRs and perfectly matched layer (PML) for the front and back boundaries parallel to the plane of the SRRs. A continuous THz wave with a linear polarization illuminates the structure at normal incidence, as shown in Fig. 1a. Cu was used as the SRR material with a conductivity of 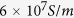 and the rings were placed on a Si substrate with ε_r_ = 11.7. The thickness of the Cu was 200 nm, while the Si substrate thickness was varied to estimate the metamaterial effective thickness t_m_.

The metamaterial arrays were fabricated using standard lift-off photolithography on thin (400 μm ± 20 μm) and thick (≈2 mm) Si substrates. Details of fabrication can be found in our previous related work^51^.

Using terahertz time domain spectrometry (THz-TDS), complex transmission spectra through the samples were obtained, including both magnitude and phase information of transmitted signal. In order to compare experimental data with simulation results, a transmission ratio was used by dividing the measured transmission data through the metamaterial samples by the transmission through the corresponding Si substrate used as a reference. However, for phase comparison, an air scan without sample was performed as the reference, following which the resulting measured phase spectrum of this reference and that of the metamaterial sample were subtracted from each other to obtain the phase of the transmission (see details in Methods).

Figure 1b–d show the simulated transmission spectra and Fig 1e–g display the corresponding measured spectra of samples R4C1, R4C3, and R1C4, respectively. Simulations and experimental measurements are in very good agreement. A sharp Fano resonance at 0.580 THz, as depicted in Fig 1b,e, originates from the smallest blue SRR (inset of Fig. 1b) through direct coupling of the SRR to the incident electric field. Interestingly, this Fano resonance arises from the excitation of the second order dipole moments in the symmetric SRR, unlike previously reported Fano metamaterials where the Fano lineshape was either due to the asymmetry of the meta-atom or interaction between symmetric meta-atoms (see Discussion). To realize an EIT-like transmission and achieve slow light, the design of R4C1 is modified to include other larger SRRs (red or green rings), as depicted in the insets of Fig. 1c,d, to create a Lorentzian resonance spectrally close to the Fano resonance and at a lower frequency. The Lorentzian resonances arise from direct coupling of these larger SRRs to the incident electric field through excitation of the second order dipole moments. Simulated transmission spectra in Fig. 1c and d and the corresponding measurements in Fig. 1f,g show that an EIT-like transmission window is thus established between the bright resonances, while no dark resonator was used in the structures. It is worth mentioning that the green and red large SRRs in R1C4 possess very close resonance frequencies and thus the observed Lorentzian resonance of R1C4 at 0.424 THz in Fig. 1d,g is the combination of both of them. However, one can use exactly identical large SRRs in both double split ring resonators (DSRRs), either two red or two green, to create a Lorentzian resonance and achieve EIT-like transmission (See discussion).

**Figure 2:**
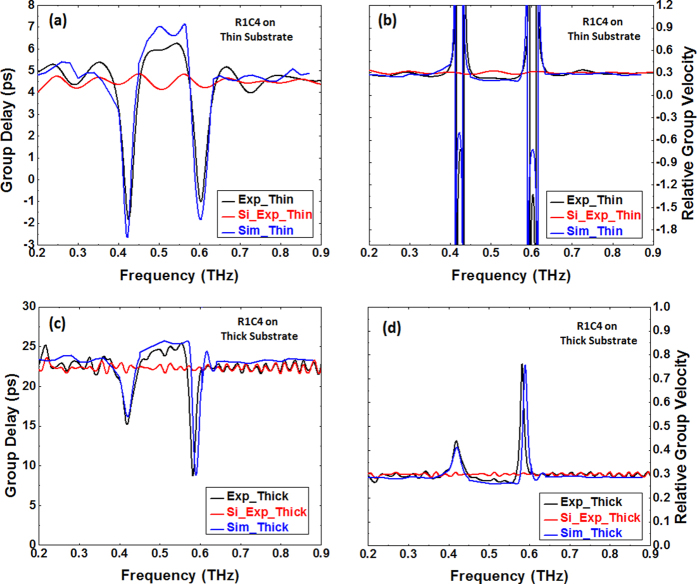
Comparison between measured and simulated group delays and velocities of sample R1C4. Group delay versus frequency for sample R1C4 on thin (**a**) and thick (**c**) substrate. Relative group velocity versus frequency for the same sample on thin (**b**) and thick (**d**) substrate. Black and blue plots represent measurement and simulation, respectively, while red plots are the measured group delay or relative velocity of light in the bare Si with the corresponding thickness.

We then determined the group delays associated with those designs (R1C4 and R4C3) which exhibited EIT-like transmission spectra. The phases of the waves transmitted through a sample and air were measured and subtracted from each other to obtain the phase difference between the two exterior surfaces of the sample (see Methods). The group delays through the samples were then determined using


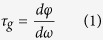


where φ is the measured phase difference and ω is the angular frequency. For example, the black plot in Fig. 2a shows the measured group delay for the R1C4 metamaterial design on 400 μm Si substrate. Simulations were performed for both designs on substrate thicknesses (t_Sub_) ranging from 50 up to 400 μm, from which group delays were similarly calculated. In doing so, we found that, as long as t_Sub_ was larger than 250 μm, the group delay spectrum obtained from simulation for a certain substrate thickness t_Sub_ matched the experimentally measured one for the same structure on 400 μm Si substrate very well (e.g. Fig. 2a for R1C4) if an extra phase delay of


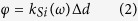


was added to the phase difference obtained from simulation, where *k*_*Si*_(*ω*) is the frequency dependent wave vector in Si and ∆*d* = (400 μm − t_Sub_) is the difference in substrate thickness between simulation and sample. By contrast, when t_Sub_ was smaller than 250 μm, the group delay spectra from simulation and experimental measurements do not match following the same procedure. This means that the contribution of the thickness of the Si substrate beyond 250 μm to the structure group delay arises only from the intrinsic property of pure silicon (i.e. *k*_*Si*_(*ω*)), whereas the portion of the Si up to 250 μm is intimately associated with the metamaterial response and influences the EIT-like resonance. In other words, the response of the metamaterial structure to an incident electromagnetic wave is not solely dependent on the thickness of metallic design (e.g. SRR) but is strongly influenced by the substrate it is deposited on. To further verify our hypothesis, we determined the group delay spectra for the R1C4 and R4C3 designs on 250 μm Si substrate after adding an extra phase delay of


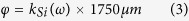


and obtained the expected near perfect match with experimental measurements on 2 mm substrate (Fig. 2c). This thickness of 250 μm therefore represents the metamaterial effective thickness, a fundamental parameter of such types of structures that has remained difficult to determine until now. And the process of matching experiment and simulation outlined here shows how to estimate it for any given EIT-like metamaterial structure.

The relative group velocities of the electromagnetic wave inside the samples were evaluated using


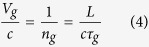


where *V*_*g*_ is the group velocity, *c* the speed of light in vacuum, *n*_*g*_ the group refractive index, *τ*_*g*_ the group delay, and *L* the sample thickness. As anticipated, a very good match between simulations and experimental measurements was obtained for both R1C4 (Fig. 2b,d on 400 μm and 2 mm substrates) and R4C3 (not shown).

**Figure 3:**
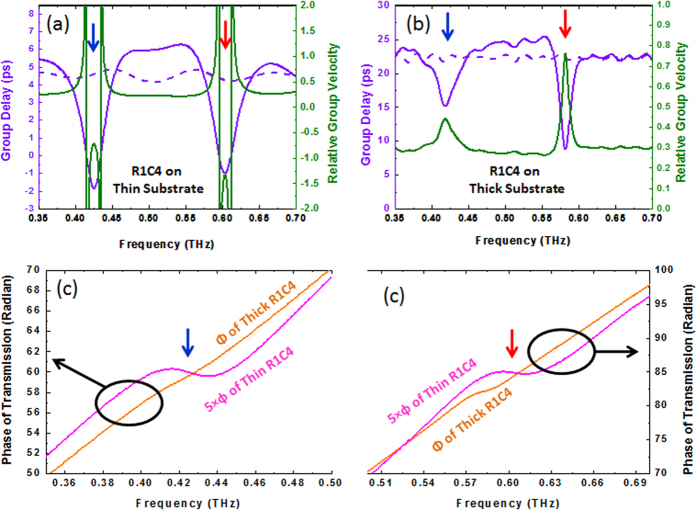
The Effect of substrate thickness on phase of transmission and group delay and velocity. (**a**) Group delay of thin Si (dashed purple), group delay of thin R1C4 sample (solid purple) and its relative group velocity (solid green). (**b**) Group delay of thick Si (dashed purple), group delay of thick R1C4 sample (solid purple) and its relative group velocity (solid green). (**c**) phase of transmission for sample R1C4 with thin (pink scaled by 5) and thick (orange) Si substrates. In all plots, the blue and red arrows indicate the positions of the Lorentzian and Fano resonances, respectively.

Figure 2b,d show that, at frequencies far away from the resonances, both thin and thick samples behave just as a bare pure Si wafer with the corresponding thickness in terms of relative group velocity, which is equal to ≈0.29 and corresponds to the inverse of the refractive index of Si (n_g_ = 3.4) in agreement with Eq. (4). At the resonance frequencies of 0.424 THz and 0.580 THz for the R1C4 design on thin substrate (Fig. 3a), the group delay (resp. velocity) undergoes sharp changes such that both zero (resp. infinite) and negative (resp. negative) values are observed. The negative group velocity is a result of anomalous dispersion in the phase spectrum created by the metamaterial structure. By contrast, in the thick sample (Fig. 3b), the group delay and group velocity remain positive in the whole spectrum, although fast light is still observable at resonances. The absence of a negative group delay or group velocity is due to the increased propagation path due to the thicker Si wafer, which smoothens the overall phase dispersion. This is clearly visible in Fig. 3c, which compares the phase of the transmission coefficient near both resonances and shows how the anomalous dispersion is smoothened in the thicker sample. In the spectral region between the resonances (0.424–0.580 THz), slower light is observed (Fig. 3a,b), with a delay of 1.5–2 [ps] compared to bare Si substrate used as a reference (dashed purple).

### The effect of spectral spacing between bright resonances on slow light

**Figure 4:**
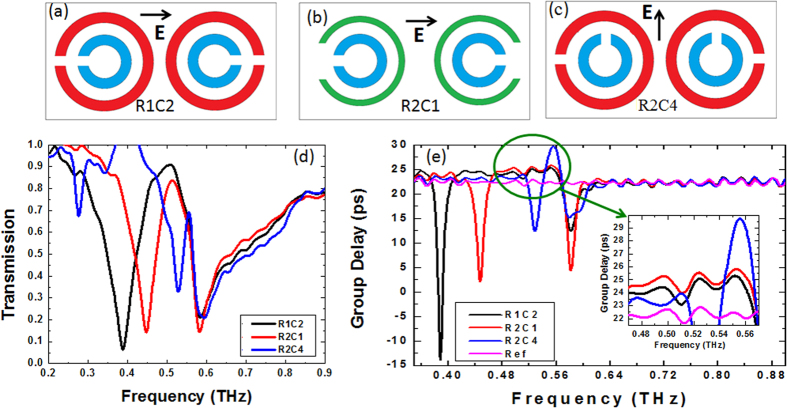
The effect of spectral spacing on slow light. (**a–c**) Schematic representations of three different structures (R1C2, R2C1, and R2C4) with coinciding Fano resonance (originating from the smaller SRRs) and progressively shifted Lorentzian resonance. (**d**) Measured transmission spectra of R1C2 (dark), R2C1(red), and R2C4 (blue). (**e**) Measured group delays, along with the bare Si substrate group delay (pink) as the reference.

After successfully determining the effective thickness of metamaterial and the impact of substrate thickness on group velocity, we further studied the effect of the separation between the two bright resonances on the extent that light is slowed between them. For the sake of this study, we designed and fabricated three additional structures on thick Si wafer such that their Fano resonance coincided at 0.580 THz, but with the lower frequency Lorentzian resonance –which originates from the larger ring– progressively shifted. Figure 4a–c depict these structures, called R1C2, R2C1, and R2C4, with associated dimensions illustrated in Fig. 1a and their measured transmission spectra shown in Fig. 4d in black, red, and blue colors, respectively. In these designs we have used either red or green large SRRs (instead of using one of each as we did in R1C4) in the unit cell to create the associated Lorentzian resonance at different frequencies. EIT-like transmission is seen for all of them between the two bright resonances, and the correspondingly measured group delays are shown in Fig. 4e along with the delay due to the bare Si wafer. The spacing between the resonance frequencies for R2C4 is smaller than for R2C1, which are both smaller than for R1C2. A comparison of the group delays τ_x_ in the spectral region between 0.54 and 0.57 THz shows that τ_R2C4_ > τ_R2C1_ > τ_R1C2_ > τ_Si_Ref_, which demonstrates that a narrower spectral spacing between bright resonances leads to a higher group delay in EIT-like transmission window between them.

## Discussion

**Figure 5:**
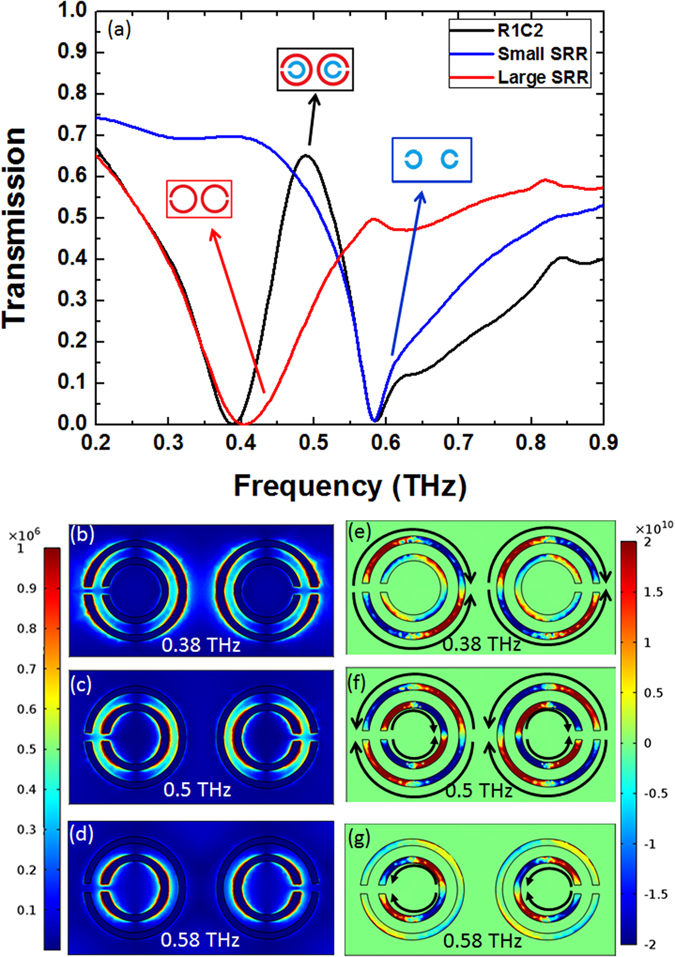
Interpretation of Fano resonance and EIT-like transparency. **(a**) Comparison of transmission spectrum of large SRR, small SRR, and R1C2 composed of both SRRs. (**b–d**) Electric field profile of sample R1C2 at resonance frequencies of 0.380 THz, 0.500 THz, and 0.580 THz, respectively, with associated current densities (J_y_ component) in (**e–g**), added black arrows illustrate more clearly the currents directions in the SRRs.

To gain a better understanding of the fundamental origin of such EIT-like transmission, we simulated and analyzed the transmission spectrum of sample R1C2. Figure 5a displays the transmission spectra obtained from the smaller SRRs, the larger SRRs and sample R1C2 which combines both. We see that the direct coupling of the incident electric field to the smaller SRRs gives rise to the Fano response (blue) at 0.580 THz, while coupling to the larger SRRs leads to the Lorentzian response (red) at 0.400 THz. Combining them to construct sample R1C2 results in a slight red-shift of the Lorentzian resonance from 0.400 THz to 0.380 THz, a narrowing of both resonances –particularly the Lorentzian– in the spectral range between them (0.380 THz < f < 0.580 THz), and a broadening of the Fano resonance at frequencies higher than 0.580 THz (black).

The electric field distributions in the plane of the R1C2 metamaterial ring arrays are shown in Fig. 5b–d at frequencies of 0.380 THz (Lorentzian resonance), 0.500 THz, and 0.580 THz (Fano resonance) respectively, along with the associated current density distributions (J_y_ in-plane component) in Fig. 5e–g where black arrows are added to better illustrate the current directions in SRRs.

At 0.380 THz (Fig. 5b), two dipole moments are created in the larger SRRs, which gives rise to the Lorentzian resonance at that frequency. There is only a slight interaction between the smaller and larger SRRs, which can be discerned by the current distributions in Fig. 5e. This interaction induces a weak current in smaller SRR (in the outer circumference) in an opposite direction of that in the larger SRR. The frequency level scheme of two coupled dipoles can explain well the aforementioned slight red shift of the Lorentzian resonance of larger SRR in R1C2^52^,^53^. Since the dipole moments in the smaller and larger SRRs are in opposite directions, the restoring force between them is diminished, which leads to the frequency red-shift of the resonance peak associated with the larger SRRs.

At 0.580 THz (Fig. 5d,g), the dipole moments and currents are primarily found in the smaller SRRs, which are thus responsible for the Fano resonance. To verify that the Fano resonance here is neither due to an asymmetry in these SRRs nor a result of interaction between them unlike prior Fano resonance reports, we performed two additional simulations: one containing just one individual small SRR and another simulation containing just one large SRR (i.e. two separate simulations, one for a small blue ring and another for large red ring of Fig. 4a). The resulting transmission spectra were exactly the same as the blue and red plots shown in Fig. 5a. We believe that such a Fano resonance is achievable in our structure, even though there is no asymmetry or interaction between SRRs, because our SRRs have a larger width relative to their radius. At resonance, the inner and outer perimeters of the SRRs equally contribute to the resonance. However, at frequencies slightly higher than the resonance the inner perimeter contributes more, while the outer perimeter is responsible for the part of the spectrum slightly lower than the resonance. This characteristic is valid for both the larger and smaller SRRs. But the dipole moments in the inner circumference of the smaller SRRs are closer to each other than those of the larger SRRs. As such, interaction between the dipole moments in the smaller SRR is stronger than for those in the larger SRR. Furthermore, the dipole moments are parallel to each other. As in the case of stereometamaterials^52^ where parallel dipoles with the same resonance frequency lead to degeneracy, the strong interaction of parallel moments in the metamaterial structures here results in splitting and hence broadening of the transmission at frequencies higher than the resonance, which results in a tail (blue curve) in the transmission spectrum and thus a Fano type resonances at 0.580 THz.

In the case of the larger SRR, the transmission (red plot in Fig. 5a) does not rise back up evenly after its resonance, for frequencies beyond 0.550 THz. More analysis by using electric field distribution confirmed the presence of higher order resonances in the larger SRR in that part of the higher frequency spectrum. As a result, when the larger SRR is combined with a smaller one to make the R1C2 design, the higher order resonances lead to an additional slight broadening of the Fano resonance tail at higher frequency, as seen in Fig. 5a (comparing black and blue plots).

**Figure 6:**
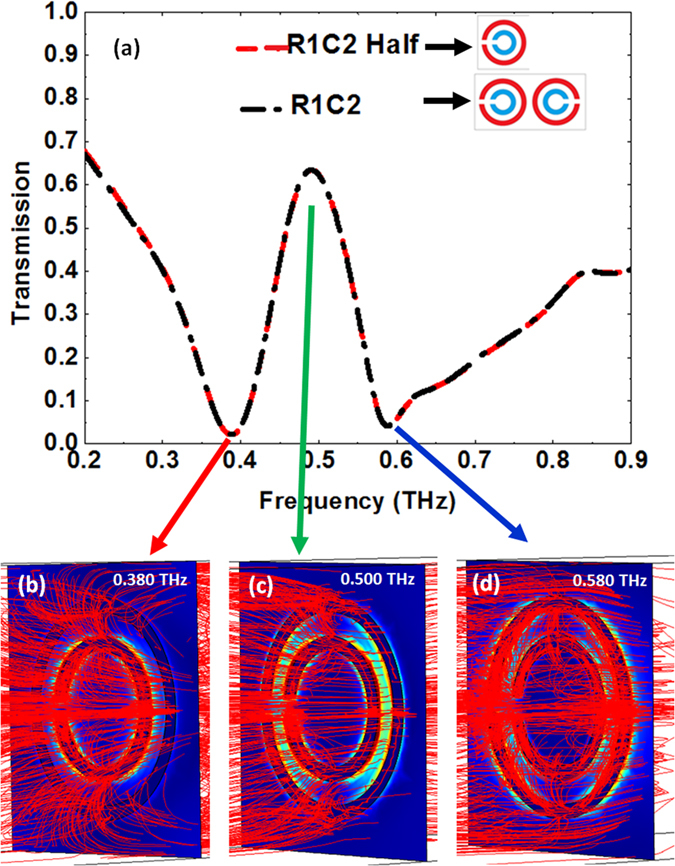
Comparison of transmission spectrum of R1C2 design with two DSRRs and one DSRR in the unit cell and demonstration of power flow across the plane of DSRR for R1C2 half at three different frequencies. (**a**) Dashed red line are transmission spectrum for R1C2 design with one DSRR in its unit cell (labeled as R1C2 Half) and dotted-dashed line is transmission spectrum for R1C2. (**b–d**) Power flow (red lines) at resonance frequencies of (**b**) 0.380 THz, (**d**) 0.580 THz and (**c**) transmission window 0.500 THz.

Eventually, at 0.500 THz, in the transmission window between the two resonances, the electric field profile shows that both SRRs are excited, but the associated dipole moments are found on the inner circumference of the larger SRR and on the outer circumference of the smaller SRR. The currents generated in the SRRs possess similar strength but are in opposite directions, which leads to destructive interference of the reflected waves out of SRRs and therefore the emergence of a transmission window at 0.500 THz. It is worth mentioning that a simulation of R1C2 with a half of its original unit cell (i.e. just one DSRR in the unit cell) gives rise to the exact same transmission spectrum as R1C2 (Fig. 6a), which demonstrates lack of interaction between two adjacent DSRRs. Thus, opening of an EIT-like window stems solely from the interaction between the small and large SRRs in each DSRR and not from interaction between two adjacent DSRRs.

The underlying mechanism behind group delay of EIT-like phenomenon in our designs can be analyzed both macroscopically and microscopically. From macroscopic point of view, an increase of group refractive index in EIT spectrum results in an increase in the optical density of the metamaterial. Like optically dense bulk materials with a high refractive index (such as Si), the incident THz radiation experiences a decrease in its velocity while transmitting through it. In order to gain a microscopic insight of group delay in our design, the calculated power flow through design R1C2 (cut by half) at three different frequencies is demonstrated in Fig. 6b–d. From the figure, it is observed that in the transmission window (0.500 THz), the THz energy is mostly confined in a small region between two SRRs while at the resonance frequencies of 0.380 THz and 0.580 THz the energy is relatively distributed over a large area. Trapping the THz radiation inside a small area between two SRRs with out-of-phase current flows and then transmitting through that small region could be one possible reason for the group delay observed in our structure^40^. Nonetheless, further and thorough study is required to better understand the mechanism of group delay in EIT metamaterials.

## Methods

### Numerical Simulation

**Figure 7:**
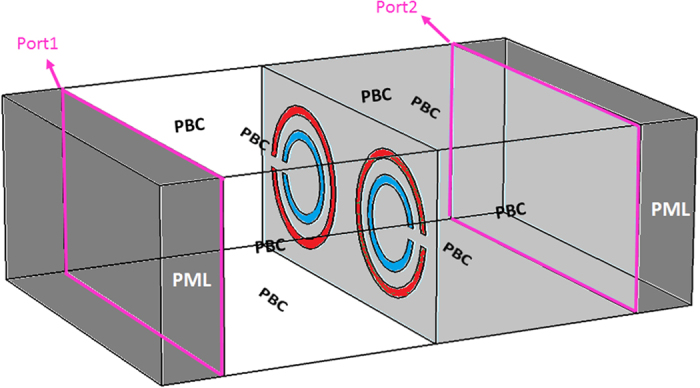
Schematic illustration of simulation environment and conditions. Light gray area is Si and white area contains air.

Numerical simulation was performed by FEM using Comsol Multiphysics software. Figure 7 shows a schematic representation of the structure under simulation. PBC was applied for all side boundaries and PML was applied for the front and back boundaries. Therefore, the metamaterial structure could be considered to be at the interface between two semi-infinite spaces of air and Si. To determine transmission and phase changes, we applied port boundaries at desired positions inside air and Si to obtain the scattering parameters of the electromagnetic field. At the input port (1) a continuous incident field with a linear polarization was launched and at the output port (2) the required scattering parameter was extracted. The transmission was calculated through T = |S_21_|^2^ where S_21_ is the scattering parameter between input and output ports depicted in the figure. Various mesh sizes were employed for different locations in the simulation depending on their refractive indices in order to satisfy the convergence condition, and the total number of mesh elements was 151, 547. In order to determine the effective thickness of metamaterial and be able to compare simulation and experimental measurements, the phase change due to air was subtracted from that obtained from the ports (φ_21_) in the aforementioned manner, and an extra phase associated with the extra Si between the thickness in the simulation and that in the experiments was considered. Therefore, the phase change of the metamaterial structure in simulation was calculated by:


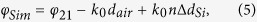


Where φ_21_ is the phase difference between the ports obtained from the scattering parameter, k_0_ is the wave number in air, d_air_ is the thickness of the space in the volume containing air, n is the Si refractive index, and ∆d_Si_ is the difference between the Si thicknesses in simulation and experiments.

### Measurement

For measurement, a THZ-TDS system was utilized in transmission mode. A schematic illustration of the measurement setup is seen in Fig. 7. A 780 nm pump laser beam (pump) with repetition rate of 65 MHz excites a photoconductive antenna (PCA) to generate linearly polarized THz wave. An electro-optical sampling scheme is used for detection such that both transmitted THz wave through the sample and a 780 nm beam meet each other at a birefringent ZnTe crystal by controlling the timing between them through a delay stage. The THz wave induces different refractive indices along two orthogonal optical axes in the crystal, which affects the polarization of the time-delayed (probe) 780 nm laser beam that passes through it. Two photodiodes are subsequently used to measure the intensity difference between these polarizations in a synchronous detection scheme by means of a voltage that represents the magnitude of the THz wave incident on the ZnTe crystal.

**Figure 8:**
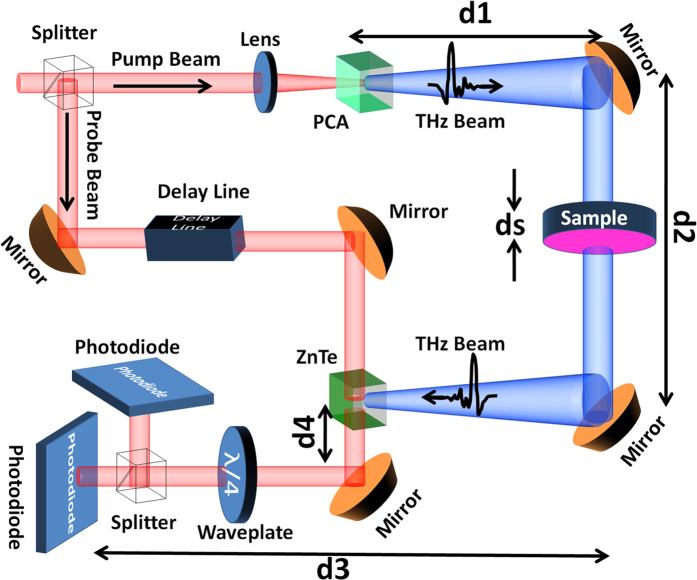
Schematic representation of THz-TDS setup used to characterize samples.

To obtain the transmission ratio, the transmitted THz wave signals thus measured through the samples were divided by that obtained through a piece of the same (but bare) Si substrate wafer used as a reference. To determine the phase difference between the exterior surfaces of the samples (φ_Sample_), the phase change between the incident THz wave and the detected THz wave transmitted through sample (φ_measure_) was measured first. Then, the phase of air and associated optical elements such as lenses or mirrors (φ_mirrors_) was subtracted according to:


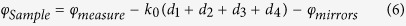


where k_0_ is the wave number of air and d_1_ + d_2_ + d_3_ + d_4_ are the distances between the PCA and the photodiodes (Fig. 8). Therefore, an air scan was required to be performed as the reference and the corresponding phase change φ_AirScan_ was measured, which includes 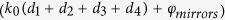. This phase was then subtracted from that of sample (φ_measure_). However, when doing so, we are also subtracting a phase change corresponding to the contribution of air with the same thickness as the sample (d_s_). Therefore, a phase change equal to k_0_d_s_ is added back, which results in the total phase change through the metamaterial sample to be:


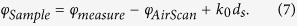


## Additional Information

**How to cite this article**: Hokmabadi, M. P. *et al.* Impact of Substrate and Bright Resonances on Group Velocity in Metamaterial without Dark Resonator. *Sci. Rep.* **5**, 14373; doi: 10.1038/srep14373 (2015).
